# Supplementary figures and images for: Genetic identification of bat species for pathogen surveillance across France
Source: PLoS One. 2022 Jan 4;17(1):e0261344. doi: 10.1371/journal.pone.0261344 (PMC8726466; doi:10.1371/journal.pone.0261344)

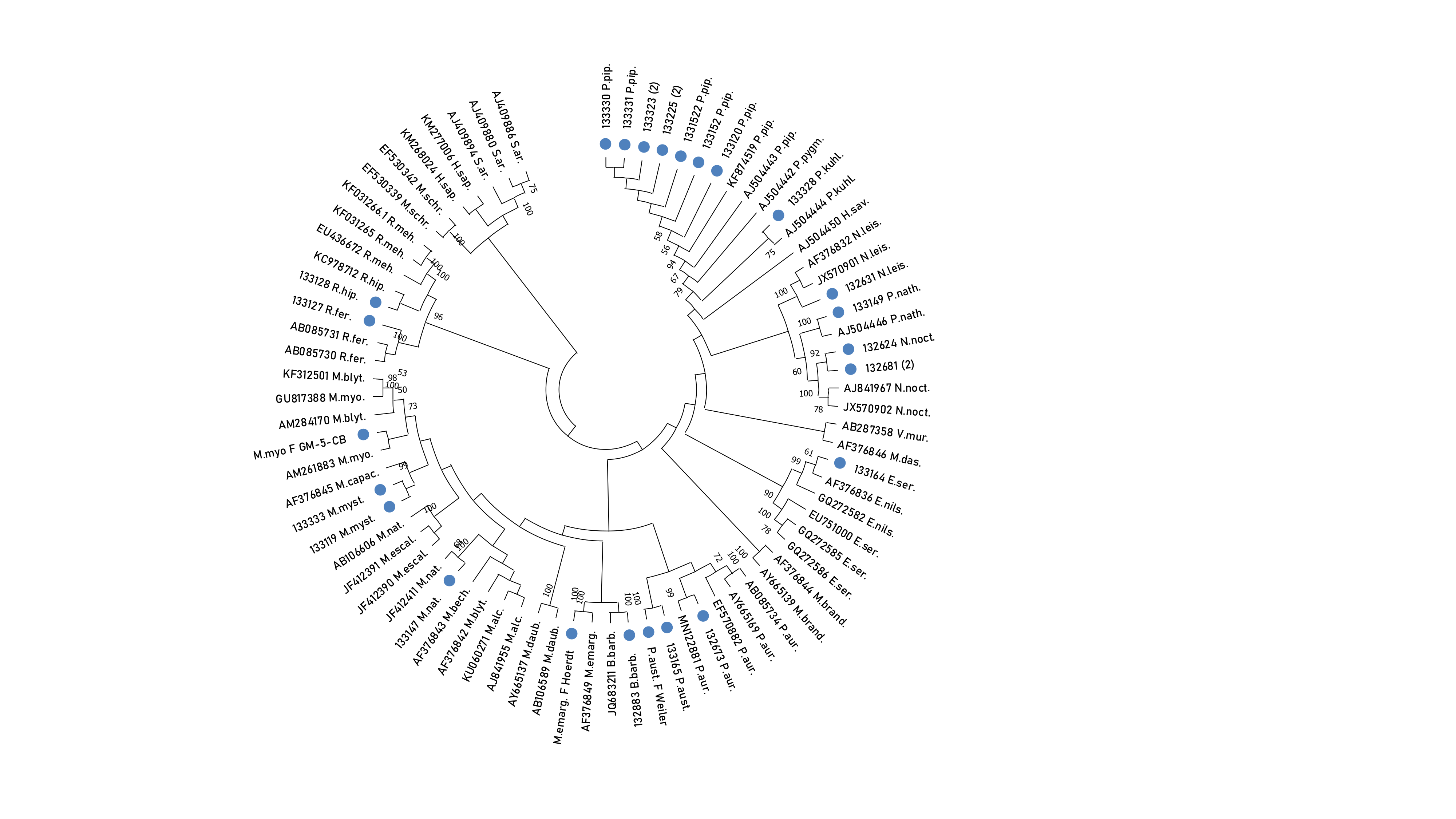

Supplement: S1 Raw images — (TIF) [file pone.0261344.s002.tif]
