# Supplementary material for: Genetic identification of bat species for pathogen surveillance across France
Source: PLoS One. 2022 Jan 4;17(1):e0261344. doi: 10.1371/journal.pone.0261344 (PMC8726466; doi:10.1371/journal.pone.0261344)

PLOS ONE

Genetic identification of bat species for pathogens surveillance across France

--Manuscript Draft--

**Manuscript Number:**	PONE-D-21-14577
**Article Type:**	Research Article
**Full Title:**	Genetic identification of bat species for pathogens surveillance across France
**Short Title:**	Species bat identification
**Corresponding Author:**	Evelyne Picard-Meyer<br>Anses<br>MALZEVILLE, FRANCE
**Keywords:**	Bat, guano, species, identification, Cytochrome B, PCR
**Abstract:**	With more than 1400 chiropteran species identified to date, bats comprise one-fifth of all mammalian species worldwide. Many studies have associated viral zoonoses with 45 different species of bats in the EU, which cluster within 5 families of bats. For example, the Serotine bats are currently shown infected by European Bat 1 Lyssavirus throughout Europe while Myotis bats are shown infected by coronavirus, herpes virus and paramyxovirus. Correct molecular host species identification is so of great importance to increase the knowledge about the ecology and evolutionary pattern of bat viruses in the EU. Bat species identification is commonly determined using morphological keys. Morphological determination of bat species from bat carcasses can be limited in some cases, due to the state of decomposition or nearly indistinguishable morphological features in juvenile bats and can lead to misidentifications. The overall objective of our study was to identify insectivorous bat species using molecular biology tools with the amplification of partial cytochrome b gene of mitochondrial DNA. Two types of sample were tested in this study, bat wing punches and bat faeces. A total of 167 bat wing punches representing 22 species, and 32 faecal pellets representing 7 species were included in the study. From the 167 bat wing punches tested, a total of 159 were genetically identified from amplification of the partial cyt b gene. All 32 faecal pellets were genetically identified based on the cyt b gene. A comparison between morphological and genetic determination showed 21 misidentifications from the 167 wing punches, representing 12.5% of misidentifications of morphological determination compared with the genetic method, across 11 species. In addition, genetic determination allowed the identification of 24 out of 25 morphologically non-determined bat samples. Our findings demonstrate the importance of a genetic approach as an efficient and reliable method to identify bat species precisely.
**Order of Authors:**	Youssef Arnaout
	Zouheira Djelouadji
	Emmanuelle Robardet
	Julien Cappelle
	Florence Cliquet
	Frederic Touzalin
	Giacomo Jimenez
	Suzel Hurstel
	Christophe Borel
	Evelyne Picard-Meyer
**Additional Information:**	
**Question**	**Response**
**Financial Disclosure**	The authors received no specific funding for this work.

Powered by Editorial Manager® and ProduXion Manager® from Aries Systems Corporation

Enter a financial disclosure statement that describes the sources of funding for the work included in this submission. Review the submission guidelines for detailed requirements. View published research articles from *PLOS ONE* for specific examples.<br><br>This statement is required for submission and **will appear in the published article** if the submission is accepted. Please make sure it is accurate.<br><br>**Unfunded studies**<br>Enter: *The author(s) received no specific funding for this work.*<br><br>**Funded studies**<br>Enter a statement with the following details:<br>• Initials of the authors who received each award<br>• Grant numbers awarded to each author<br>• The full name of each funder<br>• URL of each funder website<br>• Did the sponsors or funders play any role in the study design, data collection and analysis, decision to publish, or preparation of the manuscript?<br>• **NO** - Include this sentence at the end of your statement: *The funders had no role in study design, data collection and analysis, decision to publish, or preparation of the manuscript.*<br>• **YES** - Specify the role(s) played.<br><br>* typeset	
**Competing Interests**<br><br>Use the instructions below to enter a competing interest statement for this submission. On behalf of all authors, disclose any competing interests that could be perceived to bias this work—acknowledging all financial support and any other relevant financial or non-financial competing interests.<br><br>This statement is required for submission and **will appear in the published article** if	No authors have competing interests.

the submission is accepted. Please make sure it is accurate and that any funding sources listed in your Funding Information later in the submission form are also declared in your Financial Disclosure statement.<br><br>View published research articles from *PLOS ONE* for specific examples.<br><br>**NO authors have competing interests**<br><br>Enter: *The authors have declared that no competing interests exist.*<br><br>**Authors with competing interests**<br><br>Enter competing interest details beginning with this statement:<br><br>*I have read the journal's policy and the authors of this manuscript have the following competing interests: [insert competing interests here]*<br><br>* typeset	
**Ethics Statement**<br><br>Enter an ethics statement for this submission. This statement is required if the study involved:<br><br>• Human participants<br>• Human specimens or tissue<br>• Vertebrate animals or cephalopods<br>• Vertebrate embryos or tissues<br>• Field research<br><br>Write "N/A" if the submission does not require an ethics statement.<br><br>General guidance is provided below. Consult the submission guidelines for detailed instructions. **Make sure that all information entered here is included in the Methods section of the manuscript.**	Bats are protected species in Europe and in France. All biological samples employed in this study had been submitted for rabies diagnosis by ANSES-Nancy Laboratory for Rabies and Wildlife in accordance with the formal authorization by the French Ministry of the Environment (32). In France and within the European Union, the legal frame-work for using under experimentation purposes is governed by Regulation 2010/63/EU of the European parliament and of the council of 22 September 2010 (applicable and translated in French in 2013) and handling of wildlife animal in the field does not require any prior specific ethical approval.

**Format for specific study types**<br><br>**Human Subject Research (involving human participants and/or tissue)**<br>• Give the name of the institutional review board or ethics committee that approved the study<br>• Include the approval number and/or a statement indicating approval of this research<br>• Indicate the form of consent obtained (written/oral) or the reason that consent was not obtained (e.g. the data were analyzed anonymously)<br><br>**Animal Research (involving vertebrate animals, embryos or tissues)**<br>• Provide the name of the Institutional Animal Care and Use Committee (IACUC) or other relevant ethics board that reviewed the study protocol, and indicate whether they approved this research or granted a formal waiver of ethical approval<br>• Include an approval number if one was obtained<br>• If the study involved *non-human primates*, add *additional details* about animal welfare and steps taken to ameliorate suffering<br>• If anesthesia, euthanasia, or any kind of animal sacrifice is part of the study, include briefly which substances and/or methods were applied<br><br>**Field Research**<br><br>Include the following details if this study involves the collection of plant, animal, or other materials from a natural setting:<br>• Field permit number<br>• Name of the institution or relevant body that granted permission	
**Data Availability**<br><br>Authors are required to make all data underlying the findings described fully available, without restriction, and from the time of publication. PLOS allows rare exceptions to address legal and ethical concerns. See the PLOS Data Policy and FAQ for detailed information.	Yes - all data are fully available without restriction

A Data Availability Statement describing where the data can be found is required at submission. Your answers to this question constitute the Data Availability Statement and **will be published in the article**, if accepted.<br><br>**Important:** Stating 'data available on request from the author' is not sufficient. If your data are only available upon request, select 'No' for the first question and explain your exceptional situation in the text box.<br><br>Do the authors confirm that all data underlying the findings described in their manuscript are fully available without restriction?	
**Describe where the data may be found in full sentences. If you are copying our sample text, replace any instances of XXX with the appropriate details.**<br><br>• If the data are **held or will be held in a public repository**, include URLs, accession numbers or DOIs. If this information will only be available after acceptance, indicate this by ticking the box below. For example: *All XXX files are available from the XXX database (accession number(s) XXX, XXX.).*<br>• If the data are all contained **within the manuscript and/or Supporting Information files**, enter the following: *All relevant data are within the manuscript and its Supporting Information files.*<br>• If neither of these applies but you are able to provide **details of access elsewhere**, with or without limitations, please do so. For example:<br><br>*Data cannot be shared publicly because of [XXX]. Data are available from the XXX Institutional Data Access / Ethics Committee (contact via XXX) for researchers who meet the criteria for access to confidential data.*<br><br>*The data underlying the results presented in the study are available from (include the name of the third party*	All files are available from the Genbank database (accession number(s) MZ066766 to MZ066790).<br>All relevant data are within the manuscript and its Supporting Information files.

and contact information or URL).<br>• This text is appropriate if the data are owned by a third party and authors do not have permission to share the data.<br><br>* typeset	
Additional data availability information:	

Manuscript

Click here to access/download;Manuscript;21.05.05 Manuscript CytB.docx

Genetic identification of bat species for pathogens surveillance across France

Youssef Arnaout[1,2], Zouheira Djelouadji[2], Emmanuelle Robardet[1], Julien Cappelle[3,4], Florence Cliquet[1], Frédéric Touzalin[6], Giacomo Jimenez[5], Suzel Hurstel[7,8], Christophe Borel[5], Evelyne Picard-Meyer[1*]

1 ANSES-Nancy Laboratory for Rabies and Wildlife, WHO Collaborating Centre for Research and Management in Zoonoses Control, OIE Reference Laboratory for Rabies, European Union Reference Laboratory for Rabies, European Union Reference Laboratory for Rabies Serology, Bâtiment H, Technopole Agricole et Vétérinaire, Malzéville, France
2 VetAgro Sup Lyon Laboratory for Leptospira, Marcy l'Etoile, France
3 UMR ASTRE, CIRAD, INRAE, Université de Montpellier, Montpellier, France
4 UMR EPIA, INRAE, VetAgro Sup, Theix, France
5 CPEPESC Lorraine, Centre Ariane, 240 rue de Cumène, Neuves-Maisons, France
6 School of Biology and Environmental Science, Science Centre West, University College Dublin, Dublin, Ireland
7 GEPMA, Strasbourg, France
8 LPO Alsace, Rosenwiller, France

*Corresponding author.

Email: evelyne.picard-meyer@anses.fr (EPM)

Abstract

With more than 1400 chiropteran species identified to date, bats comprise one-fifth of all mammalian species worldwide. Many studies have associated viral zoonoses with 45 different species of bats in the EU, which cluster within 5 families of bats. For example, the Serotine bats are ~~currently shown~~ infected by European Bat 1 Lyssavirus throughout Europe while Myotis bats are ~~shown~~ infected by coronavirus, herpes virus and paramyxovirus. Correct ~~molecular~~ host species identification is ~~so of great importance to increase the knowledge about~~ the ecology and evolutionary pattern of bat viruses in the EU. Bat species identification is commonly determined using morphological keys. Morphological determination of bat species from bat carcasses can be limited in some cases, due to the state of decomposition or nearly indistinguishable morphological features in juvenile bats and can lead to misidentifications. The overall objective of our study was to identify insectivorous bat species using molecular biology tools with the amplification of partial cytochrome b gene of mitochondrial DNA. Two types of sample were tested in this study, bat wing punches and bat faeces. A total of 167 bat wing punches representing 22 species, and 32 faecal pellets representing 7 species were included in the study. From the 167 bat wing punches tested, a total of 159 were genetically identified from amplification of the partial cyt b gene. All 32 faecal pellets were genetically identified based on the cyt b gene. A comparison between morphological and genetic determination showed 21 misidentifications from the 167 wing punches, representing 12.5% of misidentifications of morphological determination compared with the genetic method, across 11 species. In addition, genetic determination allowed the identification of 24 out of 25 morphologically non-determined bat samples. Our findings demonstrate the importance of a genetic approach as an efficient and reliable method to identify bat species precisely.

Keywords

Bat, guano, species, identification, Cytochrome B, PCR

Abbreviations

LPO	League for the Protection of Birds
LRFSN	Nancy Laboratory for Rabies and Wildlife
Cyt b	Cytochrome b
n.d.	Not determined
mt	Mitochondrial
DNA	Deoxyribonucleic acid
Cox	Cytochrome c oxidase subunit 1
PCR	Polymerase Chain Reaction

Introduction

All bat species and their roosts are legally protected in France and in Europe by national and international legislation due to the significant decrease in their populations over the last few decades. Bats belong to order Chiroptera, the second largest order of mammals after rodents. With more than 1400 chiropteran species identified to date, bats comprise one-fifth of all mammalian species worldwide. Bats are divided into two sub-orders: Yangochiroptera including 12 microbat families, and Yinpterochiroptera including four microbat families in Rhinolophidea plus Old World fruit bats (1,2). Today, 51 bat species have been identified in Europe of which 35 in France, including one new cryptic species (*Myotis crypticus*) recently identified in France (3,4). All bats in Europe are insectivorous except *Rousettus aegyptiacus*, which is a frugivorous bat commonly reported in Africa, but also in Cyprus in Europe (5,6). Insectivorous bats in Europe are divided into four different families: Rhinolophidae, Vespertilionidae, Molossidae and Miniopteridae (7,8). Some studies have suggested a possible association between some bat species and micro-organisms, including more than 200 viruses, bacteria, parasites and pathogenic fungi. For example, *Myotis daubentonii*, found throughout Ireland and Europe and as far as Japan and Korea, has been shown to be infected with alphacoronaviruses, astroviruses, paramyxoviruses (9,10), lyssaviruses (11) and *Bartonella* bacteria (12). *Myotis myotis,* common in France, has been reported with the presence of alphacoronaviruses (13), herpesviruses and with *Yersinia* bacteria (14).

The role of bats in the transmission of zoonotic pathogens to both humans and animals is not clear. Their possible specific association with infectious micro-organisms highlights the importance of identifying bat species precisely to thoroughly investigate the link between the potential presence of pathogens and bat species. Species identification of individuals is generally performed by bat specialists using morphological keys (15). The morphological determination of bats is usually based on geometric morphometrics since 1980s (16–18). Since 1989, to differentiate between *M. myotis* and *M. blythii* populations, each individual has been identified with external measurements such as left and right forearm lengths, third- and fifth-digit lengths, ear width, ear length, weight, ear surface area, tragus form, number of ear folds, calcar tip form, fur colour. This set of techniques has become popular and has been used for determining bats across a large number of taxa. In some cases, morphological determination can lead to misidentification of some bat species, especially from sites harbouring multiple species belonging to the same family (19). Moreover, it is extremely difficult to determine some species precisely based on carcasses, for example *Pipistrellus pipistrellus, P. kuhlii, P. pygmaeus and P. nathusii*, or to distinguish between young *P. pipistrellus* and *P. pygmaeus* due to their similar morphological features. Finally, differences may be less obvious between these species when carcasses are in an advanced state of decomposition.

Many studies have shown the importance of genetic determination by amplification of mitochondrial DNA (mtDNA) for accurate identification of bat species (20–22). Animal mtDNA is generally a small, circular molecule containing 37 genes that are essential for normal mitochondrion function (23). Species identification and DNA barcoding have been shown to be a useful tool to better understand the relationship between the presence of pathogens and bat species. For instance, a study showed the role of bat species involved in the circulation of lyssaviruses across Canada (19,20). Genetic identification can be undertaken by testing for the partial cytochrome b gene (cyt b) (24), cytochrome c oxidase subunit 1 (cox1) gene (20) or a portion of the hypervariable domain II of the mtDNA control D-loop region that can differentiate mitochondrial haplotypes and diversity (25,26).

Interestingly, new universal cyt b primers allow species identification of 63 animal species belonging to 38 families from 14 orders and 5 classes (Mammalia, Aves, Reptilia, Actinopterygii and Malacostraca) from putrefied samples (27). Primer design is based on an alignment of referenced cyt b gene sequences (-1140 nt) from 751 Mammalia species, including bats. Primers have been used for the identification of different animal species belonging to 38 families, except bats**.** Many types of sample have been tested, including muscle, brain, lung or spleen tissue, blood, oral swabs, and others (20,27). However, the drawbacks of collecting these types of sample involve the need to capture and restrain the animals combined with the difficulty of handling them. To avoid sampling live animals, using a non-invasive sampling technique such as faeces sampling can be an alternative solution to the capture of bats. Faecal samples represent a simple and easy method to collect samples from living bats without disturbing them using capture/release methods (28,29).

One study has demonstrated the possibility of genetically identifying bats species from guano samples and other non-invasive samples based on the amplification of a segment of the mitochondrial gene cox1 (21). Despite the fact that some studies have shown disadvantages of studying faeces samples, due to the presence of PCR inhibitors, fragmented DNA and the poor quality of extracted nucleic acids (30), other studies have demonstrated the efficacy and success of studying bat guano (9,21).

The aim of this study was 1) to optimize the rapid PCR method previously described in Lopez-Oceja et al. (2016) with the new universal cyt b primers to identify autochthonous bat species from different types of bat sample, namely guano and wing punches tested for the first time; 2) to genetically determine bats in France and 3) to compare the morphological and genetic species identification of bat carcasses submitted for rabies diagnosis in 2018 and 2019.

Materials and methods

Bat specimens

The specimens used in this study were selected from a frozen and archived collection of bat carcasses submitted to the ANSES-Nancy Laboratory for Rabies and Wildlife for rabies diagnosis between 2018 and 2019. Wing punches (each ~8 mm, ~ 0.02 mg) were sampled from bat carcasses diagnosed negative for rabies and stored at -20°C. All bats were previously identified using a morphological identification key by bat specialists (15). The choice of bat samples was based on the following essential criteria: bat species and the geographic zone of collection. A total of 204 bat carcasses belonging to one of three families, Rhinolophidae*,* Vespertilionidae and Miniopteridae, representing 22 species were included in the study. Table 1 gives the characteristics of the 204 bat specimens used in this study.

In addition, bat guano (one faecal pellet ~50 mm^2; ~ 0.02 mg) was also collected by bat specialists from the French Bird Protection League (LPO) Alsace as part of authorized bat studies. Faecal pellets were collected from three different sites in the Grand Est region in France. Bat species were identified morphologically in each selected area using morphological criteria (15). A total of 32 bat faecal samples representing 7 species belonging to the families Rhinolophidae and Vespertilionidae were included in the genetic identification study (Table 2). After collection in the different sites by bat naturalists, faecal pellets were stored at -20°C and then at -80°C before analysis.

Table 1. A. Characteristics of the 167 bat carcasses included for genetic identification

Family	Bat species*	Number wing punches tested		Total
		2018	2019	2018-2019
Vespertilionidae	*Barbastella barbastellus*	1		1
	Eptesicus serotinus	1	7	8
	Eptesicus nilssonii	1		1
	Myotis bechsteinii	1		1
	Myotis brandtii		1	1
	Myotis daubentonii		3	3
	Myotis emarginatus	1		1
	Myotis myotis		11	11
	Myotis mystacinus	1	4	5
	Myotis nattereri		3	3
	Nyctalus leisleri	3	10	13
	Nyctalus noctula	2	8	10
	Pipistrellus kuhlii	4	6	10
	Pipistrellus nathusii	2	15	17
	Pipistrellus pipistrellus	1	26	27
	Pipistrellus pygmaeus	2	3	5
	Plecotus auritus	2	5	7
	Plecotus austriacus	3	6	9
	Vespertilio murinus		2	2
	Pipistrellus sp.	3	5	8
	n.d.		17	17
Rhinolophidae	*Rhinolophus ferrumequinum*		2	2
	Rhinolophus hipposideros		4	4
Miniopteridae	*Miniopterus schreibersii*		1	1
	Total of samples tested	27	140	167

n.d.: not determined

*: identification based on morphological criteria

Table 1. B. Characteristics of the 37 bat carcasses included for optimization

Family	Bat species*	Number wing punches tested		Total
		2018	2019	2018-2019
Vespertilionidae	*Barbastella barbastellus*		1	1
	Eptesicus serotinus	1	1	2
	Eptesicus nilssonii		1	1
	Myotis daubentonii		1	1
	Myotis emarginatus	1		1
	Myotis myotis	2		2
	Myotis mystacinus		2	2
	Myotis nattereri		1	1
	Nyctalus leisleri	1		1
	Nyctalus noctula	1		1
	Pipistrellus kuhlii	1	2	3
	Pipistrellus nathusii		1	1
	Pipistrellus pipistrellus		7	7
	Pipistrellus pygmaeus		1	1
	Plecotus auritus	1		1
	Plecotus austriacus	2	1	3
	Vespertilio murinus		2	2
Rhinolophidae	*Rhinolophus ferrumequinum*		1	1
	Rhinolophus hipposideros		2	2
Miniopteridae	*Miniopterus schreibersii*		3	3
	Total of samples tested			37

*: identification based on morphological criteria

Table 2. Characteristics of the 32 bat faecal specimens included in the study.

Family	Bat species*	Number bat faecal specimens 2019
Vespertilionidae	*Eptesicus serotinus*	1
	Myotis emarginatus	3
	Myotis myotis	20
	Pipistrellus pipistrellus	1
	Plecotus auritus	2
	Plecotus austriacus	1
	n.d.	1
Rhinolophidae	*Rhinolophus hipposideros*	3
	Total of samples tested	32

n.d.: not determined

*: identification based on morphological criteria

Ethics statement

Bats are protected species in Europe and in France. All biological samples employed in this study had been submitted for rabies diagnosis by ANSES-Nancy Laboratory for Rabies and Wildlife in accordance with the formal authorization by the French Ministry of the Environment (32). In France and within the European Union, the legal frame- work for using under experimentation purposes is governed by Regulation 2010/63/EU of the European parliament and of the council of 22 September 2010 (applicable and translated in French in 2013) and handling of wildlife animal in the field does not require any prior specific ethical approval.

DNA extraction

DNA extraction was performed using 1 punch per animal or 1 faecal pellet per site or per bat. Wing punches were directly used for DNA extraction, whereas a pre-extraction step was carried out to prepare bat faeces. Each faecal pellet was ground with 120 µL of 1X PBS buffer (phosphate buffered Saline, Sigma-Aldrich, Saint Quentin-Fallavier, France) then centrifuged for 5 min at 30,000 x g. For DNA extraction, 20 µL of supernatant was used and the extraction was performed using the Nucleospin Tissue Kit (Macherey Nagel, Hoerdt, France), following the manufacturer's recommendations. DNA samples were quantified using a Qubit fluorometer (Invitrogen, Marseille, France) and stored at -20°C before use.

PCR: amplification of the partial cytochrome b gene

Five µL of extracted DNA diluted to 1 ng/µL was used as template for amplification of a portion of the mitochondrial cyt b gene. We used the universal primers previously described in Lopez-Oceja et al. (2016) (forward primer L15601: 5'-TACGCAATCCTACGATCAATTCC-3' and reverse primer H15748: 5'-GGTTGTCCTCCAATTCATGTTAG-3') to amplify a 148 bp fragment of cyt b (27).

PCR amplification was performed in a 25 µL reaction volume containing 5 µL of DNA (1 ng/µL), 2.5 µL of 10X PCR Buffer without $MgCl_2$ (Invitrogen, Marseille, France), 1 µL of 50 mM $MgCl_2$, 1 µL of dNTPs (10 mM each) and 0.5 µL of Taq DNA polymerase (5 U) (Invitrogen, Marseille, France) and 1 µL of each

primer (0.4 µM). The PCR was performed with the following conditions: 3 min at 95°C, 45 cycles of 30 s at 95°C, 30 s at 48°C and 45 s at 72°C and following with a final step of extension of 5 min at 72°C.

PCR: amplification of partial D-Loop

Five µL of extracted DNA was used for PCR amplification of the hypervariable domain II of the mtDNA control D-loop region producing PCR amplicons of 424 bp. The PCR amplifications were performed in 25 µL reaction volumes using validated primers described in Moussy et al. (2015). PCR consisted of an initial denaturation step at 95°C for 3 min, followed by 45 cycles of 95°C for 30 s, 56°C for 30 s, 72°C for 45 sec, and a final extension step of 72°C for 5 min. The 25 µL reaction mixture consisted of 25 µL of DNA template diluted to 1:10, 0.5 µL of enzyme mix in 2.5 µL 10X reaction buffer, 1.0 µL of 50 mM $MgCl_2$, 1 µL of 10 mM dNTP mixture, and 1 µM of each forward and reverse primer. The D-loop primers used for PCR amplification were L-strand D-loop (5′-CTACCTCCGTGAAACCAGCAAC-3′) and H-strand D-loop (5′-CGTACACGTATTCGTATGTATGTCCT-3′).

Sequencing and phylogenetic analysis

Amplicons were analysed using 2% agarose gels stained with the intercalant SYBR Safe (Thermo Fisher Scientific, IIlkirch, France) then visualized using Bioimager (Bio-Rad, Roanne, France).

Sanger sequencing of PCR products was carried out by a service provider (Eurofins, Ebersberg, Germany) with the reverse and forward primers used in the PCR. All nucleotide sequences were assembled using Vector NTI software (version 11.5.3) (Invitrogen, France). Sequence alignments and determination of the percentages of identities and similarities were carried out with BioEdit Software (version 7.2.5) and MEGA X. Genetic identification was determined using BLAST (Basic Local Alignment Search Tool) and by constructing a phylogenetic tree with MEGA-X using the maximum likelihood algorithm and the Tamura-Nei model to calculate the distances between the 25 sequences from this study and 52 representatives of bat species (2 families, 15 species).

Results

A total of 204 bat wing punches were included in the study, 37 were included in the optimisation of the PCR and 167 were used in the PCR amplification of the partial cyt b gene by followed by sequencing of amplified products and sequence analysis.

Optimization of the cyt b PCR, D-loop PCR and sequence analysis

The cyt b and D-loop PCRs generated amplicons with the expected size of 148 bp (Fig. 1) and 424 bp (Fig. 2), respectively. The PCR amplifying cyt b was tested on DNA samples diluted to 1 ng/µL from 204 bat samples from 22 different species and 32 bat faecal samples from 7 different species. The D-loop PCR was performed on serotine bats (n=6) only. The specificity of the PCR products was confirmed for both PCRs by direct sequencing of the amplified amplicons.

Genetic identification of bat carcasses

Of 167 bat wing punches tested using cyt b PCR, 159 were genetically identified by BLAST analysis and/or phylogeny. The 159 genetically identified samples represented 22 species out of the 35 bat species reported in France. The panel of genetically identified samples included *P. pipistrellus* (n=27), *P. nathusii* (n=17), *Nyctalus leisleri* (n=13), *M. myotis* (n=11), *N. noctula* (n=10), *P. kuhlii* (n=10), *Plecotus austriacus* (n=9), *Eptesicus serotinus* (n=8), *Pipistrellus* sp. (n=8), *Pl. auritus* (n=7), *M. mystacinus* (n=5), *P. pygmaeus* (n=5), *Rhinolophus hipposideros* (n=4), *M. daubentonii* (n=3), *M. nattereri* (n=3), *Vespertilio murinus* (n=2), *R. ferrumequinum* (n=2), *Barbastella barbastellus* (n=1), *E. nilsonii* (n=1), *M. bechsteinii* (n=1), *M. brandtii* (n=1), *M. emarginatus* (n=1) and *M. schreibersii* (n=1). Of the 159 bat carcasses tested, 17 carcasses had not been previously identified with morphological criteria. The sequence analysis showed for the 17 undetermined bats the following species: *P. pipistrellus (n=9), P. kuhlii (n=6), M. mystacinus (n=1)* and *R. hipposideros (n=1).*

Eight out of the 167 samples tested by PCR could not be identified by sequence analysis of the cyt b amplicons. Interestingly, these samples had previously been morphologically determined as *E. serotinus* (n=6), *V. murinus* (n=1) and *E. nilssonii* (n=1). The partial D-loop amplification (424-bp) of five

bats morphologically identified as *E. serotinus* showed 100% of nucleotide similarity with *E. serotinus* (GenBank no. accession MF187797.1) (Fig 2).

Genetic identification of bat faeces

The cyt b PCR was optimized with the objective to amplify the unique 148 bp fragment of the cyt b gene from one faecal pellet. The cyt b PCR was performed on DNA samples extracted from 32 faecal pellet samples collected in 2019. After Sanger sequencing of the 32 amplicons, the analyses of cyt b sequences led to a specific identification of the 32 samples of bat species from one faecal pellet for the seven bat species tested. Seven species were identified genetically by sequence analysis from bat guano in this study: *M. myotis* (n=20), *M. emarginatus* (n=3), *R. hipposideros* (n=3), *P. auritus* (n=2), *E. serotinus* (n=1), *P. pipistrellus* (n=1), *P. austriacus* (n=1), and 1 was not determined (Table 2, 4).

Comparison between morphological identification and cyt b PCR analysis

The comparison between morphological and genetic identification carried out on the 167 bat samples showed the same results for 146 samples tested and identified a total of 21 misidentifications. These 21 morphological misidentifications represented 12.5% of total samples tested. The misidentifications were confirmed by bat specialists who performed a second morphological identification on these bat samples using another species determination key. Results are detailed in Table 3. Morphological identification errors were reported for 11 species: *M. brandtii* (n=1), *M. daubentonii* (n=1), *M. myotis* (n=2), *M. mystacinus* (n=2), *N. noctula* (n=1), *P. kuhlii* (n=2), *P. nathusii* (n=1), *P. pipistrellus* (n=3), *P. pygmaeus* (n=5), *P. auritus* (n=2) and *P. austriacus* (n=1). Genetic identification allowed clarifications for 24 out of 25 bats tested (17 bats morphologically identified as not determined and 4 bats morphologically identified as *Pipistrellus* sp.) (Table 3).

The phylogenetic tree shown in Figure 3 includes 52 referenced sequences extracted from the international database GenBank (Table 5) and 25 sequences of the partial cyt b gene amplified from faecal pellets (n=3) and from bat wing punches taken from bat carcasses (n=22). The nucleotide

sequences represented 15 out of the 35 autochthonous species. Sequences analyses using BLAST showed three errors of morphological determination for three following species:

- *E. nilssonii* instead of *E. serotinus;*
- *P. pipistrellus* instead of *P. pygmaeus;*
- *P. pipistrellus* instead of *M. mystacinus*.

Table 3. Results of PCR on the partial cytochrome b gene and species misidentification of bat wing punch samples compared with morphological species identification.

Family	Bat species *	Bat wing punches 2018-2019	Morphological misidentification of bat species	Clarifications	Species
Vespertilionidae	*Barbastella barbastellus*	1	0/1		
	Eptesicus serotinus	8	0/8		
	Eptesicus nilssonii	1	0/1		
	Myotis bechsteinii	1	0/1		
	Myotis brandtii	1	1/1		
	Myotis daubentonii	3	1/3		
	Myotis emarginatus	1	0/1		
	Myotis myotis	11	2/11		
	Myotis mystacinus	5	2/5		
	Myotis nattereri	3	0/3		
	Nyctalus leisleri	13	0/13		
	Nyctalus noctula	10	1/10		
	Pipistrellus kuhlii	10	2/10		
	Pipistrellus nathusii	17	1/17		
	Pipistrellus pipistrellus	27	3/27		
	Pipistrellus pygmaeus	5	5/5		
	Plecotus auritus	7	2/7		
	Plecotus austriacus	9	1/9		
	Vespertilio murinus	2	0/2		
	Pipistrellus sp.	8	0/8	7/8	*P. pipistrellus, P. kuhlii*
	n.d.	17	-	17/17	*M. mystacinus, P. pisitrellus, P. kuhlii, R. hipposideros*
Rhinolophidae	*Rhinolophus ferrumequinum*	2	0/2		
	Rhinolophus hipposideros	4	0/4		
Miniopteridae	*Miniopterus schreibersii*	1	0/1		
	Total of samples tested	167	21/167		

*: identification based on morphological criteria

Table 4. Results of PCR on the partial cytochrome b gene and species misidentification of bat guano samples compared with morphological identification.

Family	Bat species*	Nb. bat faecal specimens<br>2019	Morphological misidentification of bat species
Vespertilionidae	*Eptesicus serotinus*	1	0/1
	Myotis emarginatus	3	0/3
	Myotis myotis	20	0/20
	Pipistrellus pipistrellus	1	0/1
	Plecotus auritus	2	0/2
	Plecotus austriacus	1	0/1
	ND	1	0/1
Rhinolophidae	*Rhinolophus hipposideros*	3	0/3
	Total of samples tested	32	0/32

Table. 5: Characteristics of the partial cytochrome b gene reference sequences retrieved from GenBank and other sequences amplified from wing punches and bat guano from this study.

No.	Country	Species	Year	GenBank Accession no.	Source
1	Japan	*Rhinolophus ferrumequinum*	2003	AB085730	(33)
2	Japan	*Rhinolophus ferrumequinum*	2003	AB085731	(33)
3	Japan	*Plecotus auritus*	2003	AB085734	(33)
4	Japan	*Myotis daubentoni*	2003	AB106589	(34)
5	Japan	*Myotis nattereri*	2003	AB106606	(34)
6	Japan	*Vespertilio murinus*	2010	AB287358	(35)
7	Swiss	*Nyctalus leisleri*	2001	AF376832	(36)
8	Swiss	*Eptesicus nilssoni*	2001	AF376836	(36)
9	Swiss	*Myotis blythii*	2001	AF376842	(36)
10	Swiss	*Myotis bechsteinii*	2001	AF376843	(36)
11	Swiss	*Myotis brandtii*	2001	AF376844	(36)
12	Swiss	*Myotis capaccinii*	2001	AF376845	(36)
13	Swiss	*Myotis dasycneme*	2001	AF376846	(36)
14	Swiss	*Myotis emarginatus*	2001	AF376849	(36)
15	Cyprus	*Pipistrellus pygmaeus*	2004	AJ504442	(37)
16	Greece	*Pipistrellus pipistrellus*	2004	AJ504443	(37)
17	Macedonia	*Pipistrellus kuhli*	2004	AJ504444	(37)
18	Swiss	*Pipistrellus nathusii*	2004	AJ504446	(37)
19	Swiss	*Hypsugo savii*	2004	AJ504450	(37)
20	Swiss	*Myotis alcathoe*	2004	AJ841955	(38)
21	Swiss	*Nyctalus noctula*	2004	AJ841967	(38)
22	Spain	*Myotis myotis*	2007	AM261883	(39)
23	China	*Myotis blythii*	2006	AM284170	(40)
24	Japan	*Myotis daubentoni*	2012	AY665137	(41)
25	Japan	*Myotis brandtii*	2012	AY665139	(41)
26	Japan	*Plecotus auritus*	2012	AY665169	41
27	China	*Miniopterus schreibersii*	2004	EF530339	(42)
28	China	*Miniopterus schreibersii*	2004	EF530342	33
29	China	*Plecotus auritus*	2015	EF570882	(43)
30	Spain	*Rhinolophus euryale*	2009	EU436671	(44)
31	Spain	*Rhinolophus mehelyi*	2009	EU436672	(44)
32	Azerbaijan	*Eptesicus serotinus*	2009	EU751000	(45)
33	Russia	*Eptesicus nilssoni*	2009	GQ272582	(46)
34	Russia	*Eptesicus serotinus*	2009	GQ272585	(46)
35	Russia	*Eptesicus serotinus*	2009	GQ272586	(46)
36	Armenia	*Myotis myotis*	2009	GU817388	(47)
37	France	*Myotis escalerai*	2012	JF412390	(48)
38	France	*Myotis escalerai*	2012	JF412391	(48)
39	France	*Myotis nattereri*	2012	JF412411	(48)
40	Portugal	*Barbastella barbastellus*	2012	JQ683211	(49)
41	Swiss	*Nyctalus leisleri*	2012	JX570901	(50)

42	Greece	*Nyctalus noctula*	2012	JX570902	(50)
43	France	*Rhinolophus hipposideros*	2013	KC978712	(51)
44	Spain	*Rhinolophus mehelyi*	2014	KF031265	(52)
45	Spain	*Rhinolophus mehelyi*	2014	KF031266	(52)
46	France	*Rhinolophus euryale*	2014	KF031267	(52)
47	France	*Rhinolophus euryale*	2014	KF031268	(52)
48	Greece	*Myotis blythii*	2013	KF312501	(53)
49	Iran	*Pipistrellus pipistrellus*	2013	KF874519	(54)
50	Caucasus region	*Myotis mystacinus*	2016	KU060256	(55)
51	Caucasus region	*Myotis mystacinus*	2016	KU060257	(55)
52	Caucasus region	*Myotis alcathoe*	2016	KU060271	(55)
53	France	*Barbastella barbastellus_132883*	2019	MZ066766	This Study
54	France	*Eptesicus serotinus_133164*	2019	MZ066767	This Study
55	France	*Myotis myotis_132681*	2019	MZ066769	This Study
56	France	*Myotis mystacinus_133119*	2019	MZ066772	This Study
57	France	*Myotis mystacinus_133333*	2019	MZ066774	This Study
58	France	*Pipistresllus pipistrellus_133323*	2019	MZ066788	This Study
59	France	*Myotis nattereri_133147*	2019	MZ066775	This Study
60	France	*Nyctalus leisleri_132631*	2019	MZ066776	This Study
61	France	*Nyctalus noctule_132624*	2019	MZ066777	This Study
62	France	*Pipistrellus kuhli_133328*	2019	MZ066781	This Study
63	France	*Plecotus auratus_132673*	2019	MZ066778	This Study
64	France	*Plecotus austriacus_133165*	2019	MZ066779	This Study
65	France	*Pipistrellus nathusius_133149*	2019	MZ066782	This Study
66	France	*Pipistrellus pipistrellus_133225*	2019	MZ066773	This Study
67	France	*Pipistrellus pipistrellus_133120*	2019	MZ066783	This Study
68	France	*Pipistrellus pipistrellus_133152*	2019	MZ066784	This Study
69	France	*Pipistrellus pipistrellus_1331522*	2019	MZ066785	This Study
70	France	*Pipistrellus pipistrellus_133330*	2019	MZ066786	This Study
71	France	*Pipistrellus pipistrellus_133331*	2019	MZ066787	This Study
72	France	*Myotis myotis_132714*	2019	MZ066770	This Study
73	France	*Myotis emarginatus_Hoerdt*	2019	MZ066768	This Study
74	France	*Plecotus austriacus_Weiler*	2019	MZ066780	This Study
74	France	*Rhinolophus ferrumequinum_133127*	2019	MZ066789	This Study
76	France	*Myotis myotis_GM-5-CB*	2019	MZ066771	This Study
77	France	*Rhinolophus hipposideros_133128*	2019	MZ066790	This Study

Fig. 1. Electrophoresis gel showing the amplification of the partial cytochrome b gene (148 bp) in six bat samples.

MW= Molecular weight marker

Fig. 2. Electrophoresis gel showing the amplification of the D-loop in six *Eptesicus serotinus* bat samples.

MW= Molecular weight marker

Fig. 3. Phylogenetic tree of the partial cytochrome b (cyt b) gene of 52 referenced sequences and 25 sequences amplified from 25 samples representing 15 autochthonous bat species.

B.barb: *Barbastella barbastellus*, **E.nils**: *Eptesicus nilssonii*, **E.ser**: *Eptesicus serotinus*, **H.s:** *Homo sapiens*, **H.savi:** *Hypsugo savii,* **M.al**: *Myotis alcathoe*, **M.bech**: *Myotis bechsteinii*, **M.bly:** *Myotis blythii*, **M.br**: *Myotis brandtii*, **M.c**: *Myotis capaccinii*, **M.daub**: *Myotis daubentonii*, **M.das**: *Myotis dasycneme*, **M.em**: *Myotis emarginatus*, **M.esc**: *Myotis escalerai*, **M.myo**: Myotis *Myotis*, **M.mys**: *Myotis mystacinus*, **M. nat**: *Myotis nattereri*, **M.schr:** *Myotis schreibersii*, **N.leis**: *Nyctalus leisleri*, **N.noct**: *Nyctalus noctula*, **P.aur**: *Plecotus auritus*, **P.aust**: *Plecotus austriacus*, **P.kuh**: *Pipistrellus kuhlii*, **P.pip**: *Pipistrellus pipistrellus*, **P.pyg**: *Pipistrellus pygmaeus,* **P.nath**: *Pipistrellus nathusii*, **R.fer:** *Rhinolophus ferrumequinum*, **R.hip**: *Rhinolophus hipposideros*, **R.meh**: *Rhinolophus mehelyi*, **S.a**: *Sorex araneus,* **V.mur**: *Vespertilio murinus*.

Discussion and conclusion

To our knowledge, this study is the first to evaluate published universal primers targeting the cyt b gene (27) from two different bat matrices to genetically identify autochthonous bat species. Of the 35 species reported in France, 14 species are uncommon in France. Our study showed 12.5% of misidentification for 11 out of the 22 bat species tested. Our results corroborate the Nadin-Davis et al. (2012) study, which also showed non-negligible percentages of morphological bat species misidentification of between 10 and 15%.

It is rare and very complicated to collect samples for research or rabies diagnosis from these species. The fact that all bat carcasses included in this study came from a sample collection compiled for rabies diagnosis at ANSES Laboratory led to a relative predominance of some species: these species can pose more risk to animals and humans according to the presence of pathogens. In contrast, *P. pipistrellus* is a very common bat species in France compared with other bat species. On average, there is one *P. pipistrellus* colony in each town in France (Laurent Arthur, personal communication). *P. pipistrellus* represents on average between 45 and 50% of the total number of carcasses in the rabies diagnosis sample collection. In our study, *P. pipistrellus* represented 16% of the total number of samples. This frequency can be explained by the ecological features of bat species' distributions (possible relationship between chiropteran species' distributions and the four fundamental environmental factors of temperature, precipitation, soil and vegetation) (56).

The species could not be identified for 8 of the 167 samples tested. These samples were morphologically identified as *E. serotinus* (n=6), *E. nilssonii* (n=1) and *V. murinus* (n=1). One hypothesis of species non-identification is that the cyt b PCR was not able to identify these 8 samples due to the short amplicon length (148 bp). Two published studies investigated the genetic structure of *E. serotinus* bats by amplifying the partial D-loop region (25,26). Thus, the amplification of the partial D-loop region on the six *E. serotinus* was successful and our results on Sanger sequencing confirmed the morphological species determination as *E. serotinus*.

Regarding bat faecal specimens, results and analyses of the 32 amplicons showed that the cyt b PCR allowed specific identification of bat species from just one faecal pellet of bat guano. Bat species have previously been genetically identified from guano samples by amplification of a segment of the cox1 mitochondrial gene using real-time PCR (21). Some studies have demonstrated the advantages of using real-time PCR compared with conventional PCR : real-time PCR is more sensitive, specific and rapid as a diagnostic method for detecting *Vibrio vulnificus* and *Samonella* spp. compared with conventional PCR (57,58). Both PCR techniques are equally effective for detection of the genome of visceral leishmaniasis (59). The discrepancy between the results obtained in our study and those of the Walker et al. study likely arises from using a traditional PCR with the cyt b gene universal primers (21,27). Conventional PCR allowed us to obtain nucleotide sequences from amplicons and to genetically determine bat species using BLAST and/or phylogeny. In addition, the cost of real-time PCR is higher than conventional PCR. In our study, we demonstrated the efficacy of using universal cyt b primers to genetically identify autochthonous bats from faecal samples, a non-invasive method.

The cyt b PCR made it possible to determine 17 bat samples that could not initially be identified based on morphological criteria. Non-determination of bats can be attributed to the state of decomposition of bat carcasses, the age of the bat, especially for juveniles or pups, or inexperienced bat naturalists. Morphological identification of bat species is usually carried out on living bats. Some morphological features disappear if the carcasses are not fresh, and identification becomes more complicated, creating a source of errors. ~~Morphological identification of bat carcasses or captured bats can be different from the visual identification of living bats. In this study, morphological identification and the error rate represent only morphological identification carried out by specialists on carcasses and on captured animals, which is not the case of the visual identification of bat colonies.~~

It is important to identify bat species to preserve bats, which play a key role in the environment. Bats play an important biological and ecological role and many studies have suggested that they are reservoirs in the transmission of many zoonoses and infectious diseases from animals to humans

(3,9,60). To better understand bats and their role in the circulation of pathogens, specific and precise identification of bat species is required. Our results here showed that genetic identification is an efficient way to identify bat species in France and is a rapid and reliable tool to use compared with morphological identification.

Authors' contributions

Youssef Arnaout (YA), Zorée Djelouadji (ZD), Emmanuelle Robardet (ER), Julien Cappelle (JC), Florence Cliquet (FC), Fréderic Touzalin (FT), Giacomo Gimenez (GG), Christophe Borel (CB), Evelyne Picard-Meyer (EPM).

Conceptualization: YA, ZD, EPM

Data curation: YA, ZD, EPM

Formal analysis: YA, ZD, EPM

Investigation: YA, ZD, EPM

Methodology: YA, ZD, EPM

Project administration: YA, ZD, EPM

Supervision: ZD, EPM

Validation: YA, ZD, EPM, ER, JC, FC, FT, GG, CB

Visualization: YA, ZD, EPM, ER, JC, FC, FT, GG, CB

Writing-original draft: YA

Writing- review & editing: YA, ZD, EPM, ER, JC, FC, FT, GG, CB

Declarations of interest

None

Acknowledgments

We thank our collaborators and bat naturalists for providing the bat specimens used in this study. We also thank Alexis Petitdemange, Mélanie Badré Biarnais and Jean Luc Schereffer for their technical assistance in this study.

This work was supported by the French National Agency for Food, Environmental and Occupational Health & Safety and VetAgro Sup laboratory for Leptospira.

References

1. Lei M, Dong D. Phylogenomic analyses of bat subordinal relationships based on transcriptome data. Sci Rep. 13 2016; 6:27726.

2. Teeling EC, Springer MS, Madsen O, Bates P, O'brien SJ, Murphy WJ. A molecular phylogeny for bats illuminates biogeography and the fossil record. Science. 28 janv 2005 ;307(5709):580‑ 4.

3. Dacheux L, Cervantes-Gonzalez M, Guigon G, Thiberge J-M, Vandenbogaert M, Maufrais C, et al. A preliminary study of viral metagenomics of French bat species in contact with humans: identification of new mammalian viruses. PloS One. 2014;9(1): e87194.

4. Juste J, Ruedi M, Puechmaille SJ, Salicini I, Ibáñez C. Two New Cryptic Bat Species within the Myotis nattereri Species Complex (Vespertilionidae, Chiroptera) from the Western Palaearctic. Acta Chiropterologica. 14 févr 2019;20(2):285.

5. Hassanin A, Nesi N, Marin J, Kadjo B, Pourrut X, Leroy É, et al. Comparative phylogeography of African fruit bats (Chiroptera, Pteropodidae) provide new insights into the outbreak of Ebola virus disease in West Africa, 2014-2016. C R Biol. déc 2016;339(11‑ 12):517‑ 28.

6. Hulva P, Marešová T, Dundarova H, Bilgin R, Benda P, Bartonička T, et al. Environmental margin and island evolution in Middle Eastern populations of the Egyptian fruit bat. Mol Ecol. 2012;21(24):6104‑ 16.

7. Fenton M. Convergences in the diversification of bats. 2010;

8. Sylvia B, Guillaume M, Marc T, Petrov B, Jasja D, Lise J, et al. Action Plan for the Conservation of All Bat Species in the European Union 2018 - 2024. 2018;86.

9. Drexler JF, Corman VM, Wegner T, Tateno AF, Zerbinati RM, Gloza-Rausch F, et al. Amplification of emerging viruses in a bat colony. Emerg Infect Dis. mars 2011;17(3):449‑ 56.

10. Drexler JF, Corman VM, Müller MA, Maganga GD, Vallo P, Binger T, et al. Bats host major mammalian paramyxoviruses. Nat Commun. 24 avr 2012;3:796.

11. McElhinney LM, Marston DA, Leech S, Freuling CM, van der Poel WHM, Echevarria J, et al. Molecular epidemiology of bat lyssaviruses in Europe. Zoonoses Public Health. févr 2013;60(1):35‑ 45.

12. Stuckey MJ, Boulouis H-J, Cliquet F, Picard-Meyer E, Servat A, Aréchiga-Ceballos N, et al. Potentially Zoonotic Bartonella in Bats from France and Spain. Emerg Infect Dis. 2017;23(3):539‑ 41.

13. Falcón A, Vázquez-Morón S, Casas I, Aznar C, Ruiz G, Pozo F, et al. Detection of alpha and betacoronaviruses in multiple Iberian bat species. Arch Virol. oct 2011;156(10):1883‑ 90.

14. Mühldorfer K, Wibbelt G, Haensel J, Riehm J, Speck S. Yersinia Species Isolated from Bats, Germany. Emerg Infect Dis. mars 2010;16(3):578‑ 80.

15. Dietz C, Nill D, Helversen O. Handbook bats of Europe and Northwest Africa [Handbuch Fledermäuse Europas und Nordafrikas]. 2007;128‑ 367.

16. Adams DC, Rohlf FJ, Slice DE. Geometric morphometrics: ten years of progress following the « revolution ». Ital J Zool. 2004;71:5‑ 16.

17. Adams DC, Rohlf FJ, Slice DE. A field comes of age: geometric morphometrics in the 21st century. Hystrix Ital J Mammal. 7 mai 2013;24(1):7‑ 14.

18. James Rohlf F, Marcus LF. A revolution morphometrics. Trends Ecol Evol. avr 1993;8(4):129‑ 32.

19. Nadin-Davis S, Alnabelseya N, Knowles MK. The phylogeography of Myotis bat-associated rabies viruses across Canada. PLoS Negl Trop Dis. mai 2017;11(5):e0005541.

20. Nadin-Davis S. DNA Barcoding Facilitates Bat Species Identification for Improved Surveillance of Bat-associated Rabies across Canada. Open Zool J. 26 janv 2012;5:27‑ 37.

21. Walker FM, Williamson CHD, Sanchez DE, Sobek CJ, Chambers CL. Species From Feces: Order-Wide Identification of Chiroptera From Guano and Other Non-Invasive Genetic Samples. PloS One. 2016;11(9):e0162342.

22. Schlottau K, Eggerbauer E, Freuling CM, Beer M, Müller T, Hoffmann B. Rapid molecular species identification of indigenous bats from Germany for surveillance purposes. Infect Genet Evol J Mol Epidemiol Evol Genet Infect Dis. 2020;78:104140.

23. Boore JL. Animal mitochondrial genomes. Nucleic Acids Res. 1 avr 1999;27(8):1767‑ 80.

24. Eggerbauer E, Troupin C, Passior K, Pfaff F, Höper D, Neubauer-Juric A, et al. The Recently Discovered Bokeloh Bat Lyssavirus: Insights Into Its Genetic Heterogeneity and Spatial Distribution in Europe and the Population Genetics of Its Primary Host. Adv Virus Res. 2017;99:199‑ 232.

25. Moussy C, Atterby H, Griffiths AGF, Allnutt TR, Mathews F, Smith GC, et al. Population genetic structure of serotine bats (Eptesicus serotinus) across Europe and implications for the potential spread of bat rabies (European bat lyssavirus EBLV-1). Heredity. juill 2015;115(1):83‑ 92.

26. Troupin C, Picard-Meyer E, Dellicour S, Casademont I, Kergoat L, Lepelletier A, et al. Host Genetic Variation Does Not Determine Spatio-Temporal Patterns of European Bat 1 Lyssavirus. Genome Biol Evol. 01 2017;9(11):3202‑ 13.

27. Lopez-Oceja A, Gamarra D, Borragan S, Jiménez-Moreno S, de Pancorbo MM. New cyt b gene universal primer set for forensic analysis. Forensic Sci Int Genet. 2016;23:159‑ 65.

28. Boston ESM, Puechmaille SJ, Scott DD, Buckley DJ, Lundy MG, Montgomery IW, et al. Empirical Assessment of Non-Invasive Population Genetics in Bats: Comparison of DNA Quality from Faecal and Tissue Samples. Acta Chiropterologica. juin 2012;14(1):45‑ 52.

29. Puechmaille SJ, Petit EJ. Empirical evaluation of non-invasive capture–mark–recapture estimation of population size based on a single sampling session. J Appl Ecol. 2007;44(4):843‑ 52.

30. Taberlet null, Waits null, Luikart null. Noninvasive genetic sampling: look before you leap. Trends Ecol Evol. août 1999;14(8):323‑ 7.

31. Agreement on the Conservation of Populations of European Bats (London, 4 December 1991).

32. Ministère de l’Écologie et du Développement Durable. Arrête autorisant la capture et le transport de spécimens de chiroptères par l’Agence Française de Sécurité Sanitaire des Aliments. 2012 :3.

33. Sakai T, Kikkawa Y, Tsuchiya K, Harada M, Kanoe M, Yoshiyuki M, et al. Molecular phylogeny of Japanese Rhinolophidae based on variations in the complete sequence of the mitochondrial cytochrome b gene. Genes Genet Syst. avr 2003;78(2):179‑ 89.

34. Kawai K, Nikaido M, Harada M, Matsumura S, Lin L-K, Wu Y, et al. The status of the Japanese and East Asian bats of the genus Myotis (Vespertilionidae) based on mitochondrial sequences. Mol Phylogenet Evol. août 2003;28(2):297‑ 307.

35. Kawai K, Fukui D, Sato M, Harada M, Maeda K. Vespertilio murinus Linnaeus, 1758 Confirmed in Japan from Morphology and Mitochondrial DNA. In 2010.

36. Ruedi M, Mayer F. Molecular systematics of bats of the genus Myotis (Vespertilionidae) suggests deterministic ecomorphological convergences. Mol Phylogenet Evol. déc 2001;21(3):436‑ 48.

37. Stadelmann B, Herrera LG, Arroyo-Cabrales J, Flores-Martínez JJ, May BP, Ruedi M. Molecular Systematics of the Fishing Bat Myotis (Pizonyx) vivesi. J Mammal. 27 févr 2004;85(1):133‑ 9.

38. Stadelmann B, Jacobs D, Schoeman C, Ruedi M. Phylogeny of African Myotis Bats (Chiroptera, Vespertilionidae) Inferred from Cytochrome b Sequences. Acta Chiropterologica. 1 sept 2004;6:177‑92.

39. Stadelmann B, Lin L-K, Kunz TH, Ruedi M. Molecular phylogeny of New World Myotis (Chiroptera, Vespertilionidae) inferred from mitochondrial and nuclear DNA genes. Mol Phylogenet Evol. avr 2007;43(1):32‑48.

40. Jones G, Parsons S, Zhang S, Stadelmann B, Benda P, Ruedi M. Echolocation calls, wing shape, diet and phylogenetic diagnosis of the endemic Chinese bat Myotis pequinius. Acta Chiropterologica. déc 2006;8(2):451‑63.

41. Tsytsulina K, Dick MH, Maeda K, Masuda R. Systematics and phylogeography of the steppe whiskered bat Myotis aurascens Kuzyakin, 1935 (Chiroptera, Vespertilionidae). Russ J Theriol. 23 juin 2012;11:1‑20.

42. Tian L, Liang B, Maeda K, Metzner W, Zhang S. Molecular studies on the classification of Miniopterus schreibersii (Chiroptera: Vespertilionidae) inferred from mitochondrial cytochrome b sequences. Folia Zool. 1 janv 2004;53:303‑11.

43. Ruedi M, Csorba G, Lin L-K, Chou C-H. Molecular phylogeny and morphological revision of Myotis bats (Chiroptera: Vespertilionidae) from Taiwan and adjacent China. Zootaxa. 20 févr 2015;3920:301‑42.

44. Zhou Z-M, Guillén-Servent A, Lim B, Eger J, Wang Y-X, Jiang X-L. A new species from southwestern china in the afro-palearctic lineage of the horseshoe bats (RHINOLOPHUS). J Mammal - J MAMMAL. 1 févr 2009;90:57‑73.

45. Trujillo RG, Patton JC, Schlitter DA, Bickham JW. Molecular Phylogenetics of the Bat Genus Scotophilus (Chiroptera: vespertilionidae): Perspectives from Paternally and Maternally Inherited Genomes. J Mammal. 2 juin 2009;90(3):548‑60.

46. V. A, Bannikova A, Lebedev V, Kruskop S. Mitochondrial DNA relationships among North Palaearctic Eptesicus (Vespertilionidae, Chiroptera) and past hybridization between Common Serotine and Northern Bat. Zootaxa. 12 oct 2009;2262:40‑52.

47. Bogdanowicz W, Van Den Bussche R, Gajewska M, Postawa T, Harutyunyan M. Ancient and Contemporary DNA Sheds Light on the History of Mouse-Eared Bats in Europe and the Caucasus. Acta Chiropterologica. 1 déc 2009;11:289‑305.

48. Puechmaille SJ, Allegrini B, Boston ESM, Dubourg-Savage M-J, Evin A, Knochel A, et al. Genetic analyses reveal further cryptic lineages within the Myotis nattereri species complex. Mamm Biol. 1 mai 2012;77(3):224‑8.

49. Rebelo H, Froufe E, Brito JC, Russo D, Cistrone L, Ferrand N, et al. Postglacial colonization of Europe by the barbastelle bat: agreement between molecular data and past predictive modelling. Mol Ecol. juin 2012;21(11):2761‑74.

50. Heaney LR, Balete DS, Alviola P, Rickart EA, Ruedi M. Nyctalus plancyi and Falsistrellus petersi (Chiroptera: Vespertilionidae) from Northern Luzon, Philippines: Ecology, Phylogeny, and Biogeographic Implications. Acta Chiropterologica. janv 2012;14(2):265‑78.

51. Dool SE, Puechmaille SJ, Dietz C, Juste J, Ibáñez C, Hulva P, et al. Phylogeography and postglacial recolonization of Europe by Rhinolophus hipposideros: evidence from multiple genetic markers. Mol Ecol. août 2013;22(15):4055‑70.

52. Puechmaille S, Teeling E. Non-invasive genetics can help find rare species: A case study with Rhinolophus mehelyi and R. euryale (Rhinolophidae: Chiroptera) in Western Europe. Mammalia. 1 mai 2014;78:251‑5.

53. Ruedi M, Stadelmann B, Gager Y, Douzery EJP, Francis CM, Lin L-K, et al. Molecular phylogenetic reconstructions identify East Asia as the cradle for the evolution of the cosmopolitan genus Myotis (Mammalia, Chiroptera). Mol Phylogenet Evol. déc 2013;69(3):437‑ 49.

54. Benda P, Faizolâhi K, Andreas M, Obuch J, Reiter A, Ševčík M, et al. Bats (Mammalia: Chiroptera) of the Eastern Mediterranean and Middle East. Part 10. Bat fauna of Iran. Acta Soc Zool Bohemicae. 1 janv 2012;76:163‑ 582.

55. Benda P, Gazaryan S, Vallo P. On the distribution and taxonomy of bats of the Myotis mystacinus morphogroup from the Caucasus region (Chiroptera: Vespertilionidae). Turk J Zool. 18 déc 2015;2016.

56. Wang HG, Owen RD, Sánchez‐ Hernández C, Romero‐ Almaraz MDL. Ecological characterization of bat species distributions in Michoacán, México, using a geographic information system. Glob Ecol Biogeogr. 2003;12(1):65‑ 85.

57. Zhang G, Brown EW, González-Escalona N. Comparison of real-time PCR, reverse transcriptase real-time PCR, loop-mediated isothermal amplification, and the FDA conventional microbiological method for the detection of Salmonella spp. in produce. Appl Environ Microbiol. sept 2011;77(18):6495‑ 501.

58. Kim HS, Kim D-M, Neupane GP, Lee Y, Yang N-W, Jang SJ, et al. Comparison of conventional, nested, and real-time PCR assays for rapid and accurate detection of Vibrio vulnificus. J Clin Microbiol. sept 2008;46(9):2992‑ 8.

59. Pereira MR, Rocha-Silva F, Graciele-Melo C, Lafuente CR, Magalhães T, Caligiorne RB. Comparison between conventional and real-time PCR assays for diagnosis of visceral leishmaniasis. BioMed Res Int. 2014;2014:639310.

60. Shipley R, Wright E, Selden D, Wu G, Aegerter J, Fooks AR, et al. Bats and Viruses: Emergence of Novel Lyssaviruses and Association of Bats with Viral Zoonoses in the EU. Trop Med Infect Dis. 7 févr 2019;4(1).

Figure 1

Click here to access/download;Figure;Fig1.tif

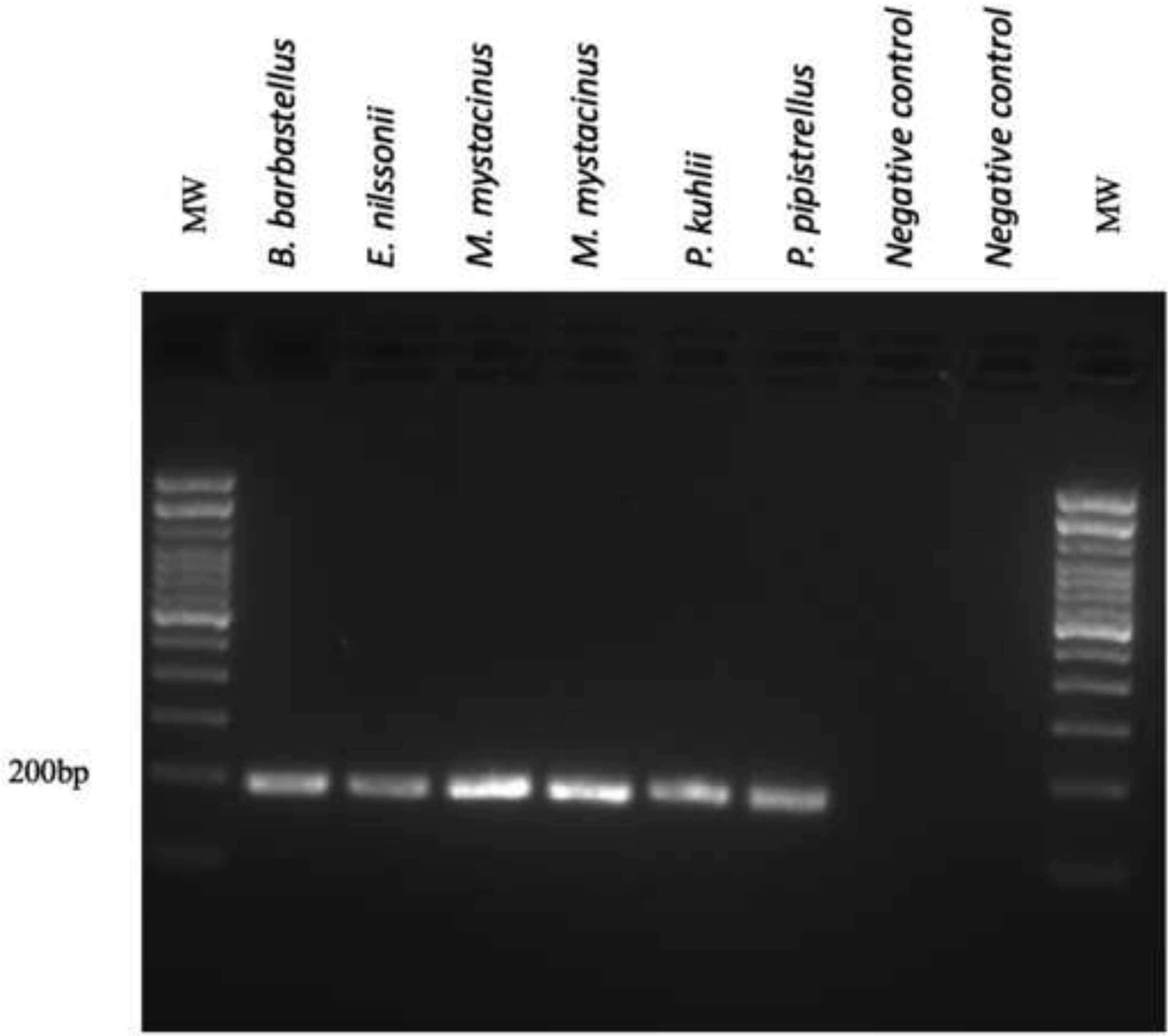

Figure 2

Click here to access/download;Figure;Fig2.tif

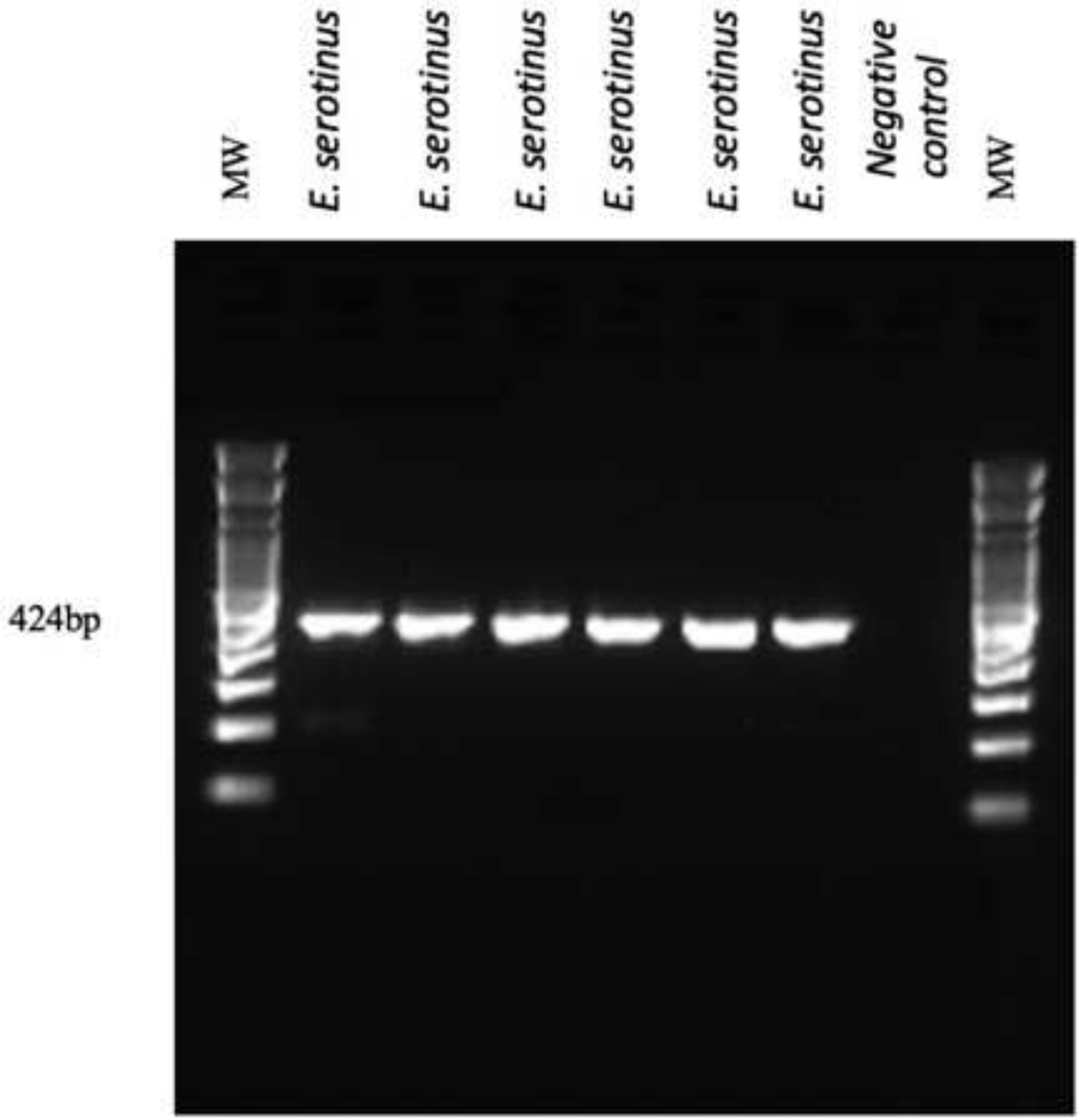

Figure 3

Click here to access/download;Figure;Fig3.tif

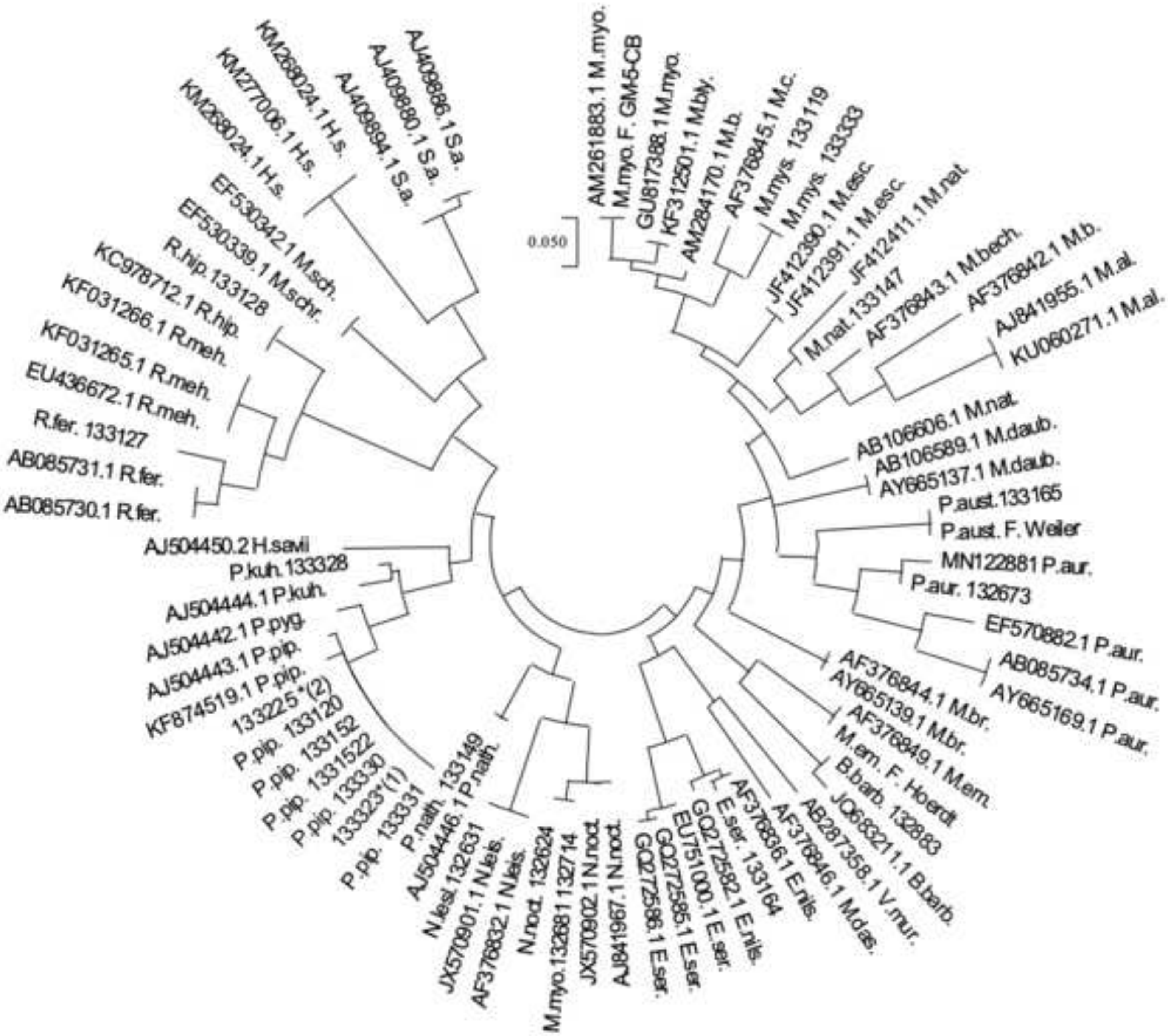

Supplement: Attachment — Submitted filename: Reviewer Comments_PONE-D-21-14577_reviewer.pdf [file pone.0261344.s003.pdf]
